# Antagonistic Interplay between MicroRNA-155 and IL-10 during Lyme Carditis and Arthritis

**DOI:** 10.1371/journal.pone.0135142

**Published:** 2015-08-07

**Authors:** Robert B. Lochhead, James F. Zachary, Luciana Dalla Rosa, Ying Ma, John H. Weis, Ryan M. O’Connell, Janis J. Weis

**Affiliations:** 1 Division of Microbiology and Immunology, Department of Pathology, University of Utah, Salt Lake City, Utah, United States of America; 2 Department of Veterinary Pathobiology, University of Illinois at Urbana-Champaign, Urbana, Illinois, United States of America; 3 Department of Microbiology and Parasitology, Universidade Federal de Santa Maria, Santa Maria, Rio Grande do Sul, Brazil; University of Kentucky College of Medicine, UNITED STATES

## Abstract

MicroRNA-155 has been shown to play a role in immune activation and inflammation, and is suppressed by IL-10, an important anti-inflammatory cytokine. The established involvement of IL-10 in the murine model of *Borrelia burgdorferi*-induced Lyme arthritis and carditis allowed us to assess the interplay between IL-10 and miR-155 *in vivo*. As reported previously, *Mir155* was highly upregulated in joints from infected severely arthritic B6 Il10^-/-^ mice, but not in mildly arthritic B6 mice. In infected hearts, *Mir155* was upregulated in both strains, suggesting a role of miR-155 in Lyme carditis. Using *B*. *burgdorferi-*infected B6, Mir155^-/-^, Il10^-/-^, and Mir155^-/-^ Il10^-/-^ double-knockout (DKO) mice, we found that anti-inflammatory IL-10 and pro-inflammatory miR-155 have opposite and somewhat compensatory effects on myeloid cell activity, cytokine production, and antibody response. Both IL-10 and miR-155 were required for suppression of Lyme carditis. Infected Mir155^-/-^ mice developed moderate/severe carditis, had higher *B*. *burgdorferi* numbers, and had reduced Th1 cytokine expression in hearts. In contrast, while Il10^-/-^ and DKO mice also developed severe carditis, hearts had reduced bacterial numbers and elevated Th1 and innate cytokine expression. Surprisingly, miR-155 had little effect on Lyme arthritis. These results show that antagonistic interplay between IL-10 and miR-155 is required to balance host defense and immune activation *in vivo*, and this balance is particularly important for suppression of Lyme carditis. These results also highlight tissue-specific differences in Lyme arthritis and carditis pathogenesis, and reveal the importance of IL-10-mediated regulation of miR-155 in maintaining healthy immunity.

## Introduction

Lyme disease (LD) is caused by infection with the tick-borne spirochete, *Borrelia burgdorferi* [1], and is the most common vector-borne disease in the United States, with approximately 300,000 cases per year [2]. LD can lead to a variety of symptoms, including rash, fatigue, fever, joint pain, neurological symptoms, carditis, and arthritis [3]. LD symptoms generally resolve after appropriate antibiotic therapy. However, symptoms such as arthritis can persist for months or years following effective spirochetal killing by oral and IV antibiotics, called treatment-refractory Lyme arthritis (LA) [4]. Lyme carditis is a rare but potentially fatal early disease manifestation in some patients [5]. It is not known why disease manifestation and severity vary so greatly in LD patients, but is likely due to a number of factors, including host genetics [6] and strain variation [7, 8].

Many clinical aspects of LD can also be observed in inbred strains of mice, including LA and carditis [9]. Some mouse strains, such as B6 mice, develop mild LA and carditis symptoms, while others, such as C3H mice, develop more severe symptoms independent of bacterial number [9]. Forward [10] and reverse genetics have also been used to identify genes associated with LA severity. IL-10 has been shown to be an important modulator of disease; Il10^-/-^ mice develop more severe LA than wild-type B6 mice, and arthritis in these mice is accompanied by high cytokine levels, low bacterial numbers, and a robust T cell response and IFNγ signature [11, 12]. Lyme carditis susceptibility and persistence are associated with increased bacterial burden in hearts in C3H [13] and immunodeficient mice [14]. Studies have also shown that Stat1 [15] and invariant NKT-derived IFNγ [16] are protective in mouse models of carditis. Thus, while rapid and robust immune activation is required to limit pathogen spread, excessive inflammation can also lead to tissue damage. It is therefore also essential for the immune response to be tightly regulated in terms of both amplitude and duration, and to fully resolve following antibiotic treatment and pathogen clearance. Many immune regulators are required in order to maintain this balance between host defense and immune response, and breakdown in one or more of these immune regulators often lead to more severe disease symptoms.

MicroRNAs (miRs) are small, noncoding RNA molecules that have recently been identified as key regulators of a variety of cellular processes [17]. Defects in miR function often lead to inflammatory pathogenesis and autoimmunity [18]. Of interest to this study, miR-155 is a highly conserved pro-inflammatory miR that is required for normal adaptive and innate immune function [19]. Importantly, elevated miR-155 expression is associated with a number of inflammatory diseases, including rheumatoid arthritis [20].

Microarray analysis performed recently identified miR-155 as being strongly upregulated in joint tissue of Il10^-/-^ mice infected with *B*. *burgdorferi* [21]. This upregulation was not observed in mildly arthritic B6 mice, nor was it observed in the severely arthritogenic C3H mouse strain. These findings suggested that IL-10 was involved in regulation of miR-155 expression in mouse joints during LA. McCoy, et al. demonstrated that miR-155 expression is down-regulated by IL-10 in macrophages in a STAT3-dependent manner [22]. IL-10 is a key anti-inflammatory cytokine and is critical in limiting a wide range of inflammatory responses through down-regulation of IFNγ signaling, inhibition of NF-κB activity, and suppression of inflammatory macrophage and neutrophil activity [23].

We hypothesized that IL-10-mediated regulation of miR-155 could be important in the clinical manifestations of *B*. *burgdorferi* infection, as others have shown several opposing immune phenotypes in mice lacking either IL-10 or miR-155. For example, as opposed to Il10^-/-^ mice, Mir155^-/-^ mice are resistant to autoimmune arthritis [24] and have impaired immune function [25, 26]. The purposes of this study were to determine to what degree the immunosuppressive activity of IL-10 was due to its down-regulation of the pro-inflammatory miR-155, and to ask what effect this IL-10/miR-155 antagonistic regulatory circuit has on balancing host defense and immune activation during infection with *B*. *burgdorferi*.

## Materials and Methods

### Ethics Statement

Mice were housed in the University of Utah Comparative Medicine Center (Salt Lake City, UT), following strict adherence to the guidelines according to the National Institutes of Health for the care and use of laboratory animals, as described in the Guide for the Care and Use of Laboratory Animals, 8^th^ Edition. Protocols conducted in this study were approved and carried out in accordance to the University of Utah Institutional Animal Care and Use Committee (Protocol Number 12–01005). Mouse experiments were performed under isofluorane anesthesia, and every effort was made to minimize suffering.

### Mice, bacterial cultures and infections

C57BL/6 (B and B6.129P2-IL10TmiCgn (*Il10* ^*-/-*^) mice were purchased from The Jackson Laboratory. *Mir155* ^-/-^ mice were generated on a C57BL/6 genetic background as described [25]. *Il10* ^-/-^ *Mir155* ^-/-^ (DKO) mice were generated by crossing *Il10* ^-/-^ with *Mir155* ^-/-^, and genotyping was performed according to protocol provided by The Jackson Laboratory for the *Il10* mutation, and as described previously for the *Mir155* mutation [25]. Mouse colonies were cared for by University of Utah Comparative Medicine Center staff in a specific pathogen free facility. Littermates were co-housed (5 mice per cage) and were monitored daily for health status. Experiments were performed using mice 6–8 weeks of age, and mice were randomly assigned into experiment and control groups. Number of mice used in each experimental group is indicated in figure and table legends, and three mice were used as a control group for each experiment. To avoid colitis development, *Il10* ^*-/-*^ and DKO mice were kept on antibiotic water (trimethoprim and sulfamethoxazole) until 1 day prior to infection. Mice were monitored daily for health status. Mice were infected with 2x10^4^ bacteria of *B*. *burgdorferi* strain N40 (a gift from S. Barthold, University of California, Davis, CA) by intradermal infection into the skin of the back. Infection was confirmed by ELISA quantification of *B*. *burgdorferi-*specific IgM and IgG concentrations. Mice were humanely euthanized by cervical dislocation after sedation using isofluorane.

### Isolation of RNA and quantitative RT-PCR

RNA was purified from heart, ankle skin, or tibiotarsal joints treated with RNA stabilization solution (Qiagen) immediately following euthanasia. Total RNA was recovered from homogenized tissue using the miRNeasy kit (Qiagen). RNA from BMDMs was recovered using Trizol reagent (Invitrogen). RNA was reverse transcribed, and transcripts were quantified using a Roche LC-480 as performed previously [27] Briefly, reactions were carried out using SYBR Green Master Mix (Roche) according to manufacturer protocols, and RT efficiency was calculated using Roche LC-480 software. Primers were tested for optimal annealing temperature and correct amplicon size prior to use, and melting curves were performed in each experiment to confirm a single product. Positive RT and negative H2O controls were included in each experiment. Primers used in this study were designed according to reagent manufacturer recommendations (Roche), and were as follows: *Mir155* FWD (5’- AAACCAGGAAGGGGAAGTGT-3’) REV (5’-ATCCAGCAGGGTGACTCTTG-3’) and *Il12a(p35)* FWD (5'-ACCAGCACATTGAAGACCTG-3') REV (5'-GACTGCATCAGCTCATCGAT-3'). Primer sequences for *B. burgdorferi 16S rRNA, Bactin, Il1b, Tnfa [27], Ifng, Cxcl9, Cxcl10,* and *Il6* [28] can be found in indicated citations.

### Bone marrow-derived macrophage stimulation

Bone marrow-derived macrophages (BMDMs) were isolated as described [29], and were plated in 12-well plates at a density of 6x10^5^/ml in Nudridoma (Roche)-supplemented media, and stimulated with live *B*. *burgdorferi* N40 at 10:1 multiplicity of infection (MOI). After 24 hours, cytokine levels in cell supernatants were analyzed by ELISA, and RNA was extracted from cells for mRNA and *Mir155* quantification by qRT-PCR.

### ELISA analysis of cell supernatant and serum

Cell supernatants and sera were used immediately or stored at -20°C prior to analysis. Cytokine concentration was detected by sandwich ELISA using capture and biotinylated antibodies against mouse TNFα, IL-1β, IL-6, IFNγ and IL-12 using antibody clones described previously [21]. *B*. *burgdorferi*-specific IgG, IgG1, IgG2c, IgG3, and IgM concentrations in serum were quantified by ELISA as performed in our lab previously using serial dilutions of infected mouse serum to determine optimal concentration for each isotype [30].

### Phagocytosis Assay

Phagocytosis activity was assayed as performed previously [31]. Peritoneal macrophages were harvested 4 days after intraperitoneal administration of 3 ml of 3% sterile thioglycolate. Macrophages were collected with ice-cold PBS, and red blood cells lysed with ACK lysis buffer. Cells in RPMI-10% FBS were plated at 5×10^5^/well in 12-well plate and allowed to adhere overnight, when non-adherent cells were removed by washing. *B*. *burgdorferi* N40 expressing GFP were added to the macrophages in RPMI.B (75% RPMI(+10% FBS)+25% BSKII(+6% rabbit serum)) at a 50:1 ratio [32, 33]. Plates were centrifuged at 500×g for 5 minutes and incubated for 1 or 2 hours at 37°C, conditions previously shown to capture midway and maximal phagocytosis [21]. Wells were washed to remove unassociated bacteria and incubated with 0.25% trypsin in RPMI for 7 minutes at 37°C to release extracellular bacteria from the macrophages prior to collecting. Trypsinized cells were washed 3 times in cold PBS, suspended in flow buffer, and analyzed using a BD LSRII flow cytometer to measure trypsin-resistant GFP bacteria within macrophages, as described [34]. Baseline fluorescence was determined for cells not receiving GFP *B*. *burgdorferi* in each treatment group.

### Assessment of arthritis and carditis severity

Arthritis and carditis severity was determined as described [21]. Arthritis was assessed at 4 weeks post-infection. Ankle measurements were obtained using a metric caliper. Rear ankle joints were prepared for assessment of histopathology by removal of the ankle skin and fixation of tissue in 10% neutral buffered formalin. Decalcified joints were embedded in paraffin, sectioned at 3 μm, and stained with H&E. Each slide was scored from 0 to 5 for various aspects of disease, including polymorphonuclear leukocyte (PMN) and mononuclear cell (lymphocytes, monocytes, macrophages) infiltration into inflammatory processes, tendon sheath thickening (hypertrophy and hyperplasia of surface cells and/or underlying dense sheets of cells resembling immature fibroblasts, synoviocytes, and/or granulation tissue), reactive/reparative responses (periosteal hyperplasia and new bone formation and remodeling), and overall lesion (composite score based on all lesions observed in 6–8 sections per joint), with 5 representing the most severe lesion, and 0 representing no lesion. Hearts were assessed for carditis by histopathologic evaluation at 2 and 3 weeks post-infection. Hearts were fixed in 10% neutral buffered formalin, embedded in paraffin and coronally sectioned at 3 μm, and stained with H&E. Scoring was performed based on a composite of 12 sections per sample. Each slide was given a score from 0 to 5 for overall lesion, PMN infiltrate, mononuclear infiltrate, and vasculitis severity (vasculitis/perivasculitis), with 5 being most severe. Ankle measurements, arthritic lesions, and carditis severity were assessed in coded samples.

### Data and statistical analysis

All graphical data represent mean ± SEM. Statistical analysis was performed using Prism 5.0c software. Multiple-sample data sets were analyzed by one-way ANOVA followed by appropriate *post-hoc* analysis, as stated in figure legends. Two-sample data sets were analyzed by Student *t-*test. Statistical significance (p<0.05) is indicated in figure legends.

## Results

### MicroRNA-155 expression is elevated in hearts of infected B6 and Il10^-/-^ mice

*B*. *burgdorferi* can localize to skin, heart, and joint tissue, which may result in erythema migrans, carditis, and arthritis, respectively. It was shown previously that miR-155 levels were elevated in infected joints of Il10^-/-^ mice at 2 and 4 weeks post-infection [21]. *Mir155* primary transcripts were also elevated in joints of Il10^-/-^ (but not B6) mice at 4 weeks post-infection **(Table 1)**. In order to determine if *Mir155* was upregulated in heart and skin tissue, infected B6 and Il10^-/-^ mice with *B*. *burgdorferi* were analyzed at 3 weeks post-infection (**Table 1**), a time point consistent with the peak for NF-κB dependent responses in *B*. *burgdorferi*-infected mice [35]. This miR was upregulated in both heart and skin tissue in infected Il10^-/-^ mice, as expected. Interestingly, *Mir155* was also upregulated in infected B6 heart tissue, a tissue known to display cytokine induction and carditis at this time point [35]. This suggested that miR-155 may be playing a unique role in regulating heart inflammation and carditis during *B*. *burgdorferi* infection. This finding, along with previous observations showing elevated levels of miR-155 in infected Il10^-/-^ joint tissue [21], and STAT3-dependent miR-155 suppression by IL-10 [22], led to the prediction that the IL-10/miR-155 regulatory circuit described by McCoy, et al. [22] might be an important mechanism of immune regulation during Lyme borreliosis.

**Table 1:** Expression of *Mir155* in hearts, ankle skin, and joints of B6 and Il10^-/-^ mice.

Strain	*Mir155* in heart		*Mir155* in ankle skin		*Mir155* in joints	
	Control	Infected	Control	Infected	Control	Infected
B6	1.8 (±0.02)	**2.3 (±0.1)**	0.1 (±0.04)	0.1 (±0.01)	0.16 (±0.01)	0.13 (±0.02)
Il10^-/-^	2.0 (±0.1)	**3.4 (±0.5)**	0.05 (±0.04)	**0.23 (±0.02)**	0.13 (±0.03)	**0.55 (±0.11)**

Mean (±SEM) *Mir155* copy number, relative to *β-actin*, in hearts, ankle skin, and joints of B6 and Il10^-/-^ mice, either uninfected control or infected with *B*. *burgdorferi* for 3 (hearts, skin) or 4 (joints) weeks. Numbers in bold indicate statistically significant difference in *Mir155* expression after infection, and were determined by Student’s t-test (p<0.05, n≥5 mice per group).

### IL-10 and miR-155 have antagonistic effects on macrophage activation

The immunosuppressive activity of IL-10 is required for maintaining healthy immunity during infection, and has pleiotropic effects on many inflammatory mediators, notably through suppressing a number of innate and adaptive cytokines including IFNγ. In contrast to IL-10, miR-155 has an enhancing effect on some of these same pathways involved in immune activation and cytokine production, including JAK/STAT signaling [19]. We therefore wanted to know to what degree IL-10-mediated suppression of miR-155 contributed to its role as an immune suppressor (miR-155-dependent), versus other (miR-155-independent) effects on Lyme disease pathogenesis. To determine this, B6 mice lacking IL-10 (Il10^-/-^) were crossed to B6 mice lacking miR-155 (Mir155^-/-^) to generate double knockout mouse lacking both IL-10 and miR-155 (Il10^-/-^ Mir155^-/-^ DKO, Material and Methods).

**Fig 1:**
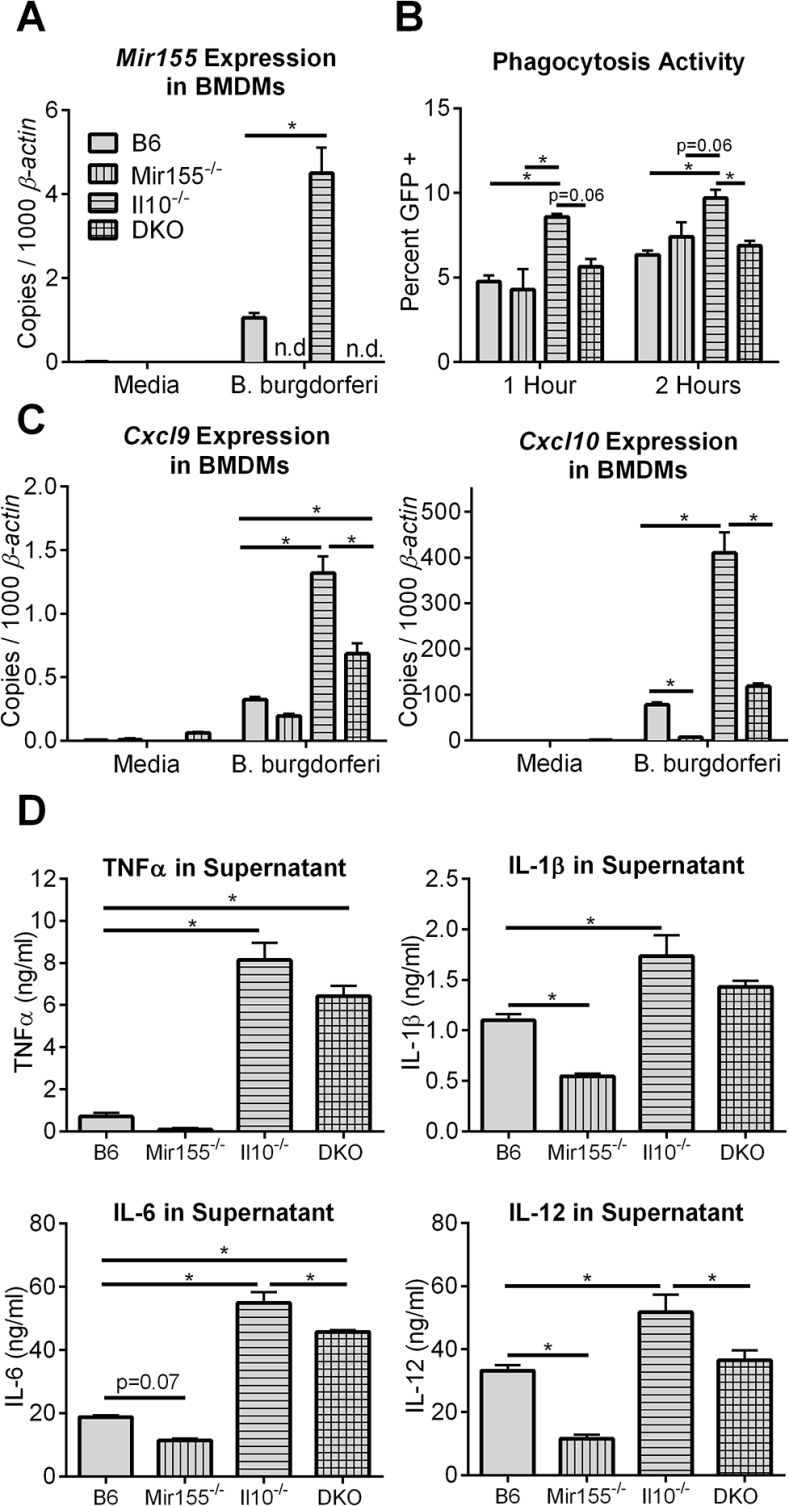
Activation of macrophages is modulated by IL-10/miR-155 regulatory network. Bone marrow-derived macrophages (BMDMs) and peritoneal macrophages were isolated from B6, Mir155^-/-^, Il10^-/-^, and DKO mice, and cells were stimulated with media alone or with *B*. *burgdorferi*. **A,** Expression of *Mir155* in BMDMs stimulated with *B*. *burgdorferi* for 24 hours by quantitative real-time PCR (qRT-PCR). **B,** Peritoneal macrophages were incubated with GFP-expressing *B*. *burgdorferi* for 1 or 2 hours, and cells containing intracellular bacteria (GFP-positive) were determined by flow cytometric analysis. **C,** *Cxcl9* and *Cxcl10* expression in BMDMs stimulated with *B*. *burgdorferi* for 24 hours, by qRT-PCR. **D,** Cytokine levels in culture supernatant from BMDMs stimulated by *B*. *burgdorferi* for 24 hours, measured by ELISA. Statistically significant differences between strains were determined by ANOVA (Tukey’s post-hoc), and are indicated (* p<0.05), with adjusted p-values included for trends not achieving statistical significance (p<0.1). Results are from one experiment (n = 3 triplicates per mouse strain).

Bone marrow-derived macrophages (BMDMs) were isolated from B6, Mir155^-/-^, Il10^-/-^, and DKO mice, and stimulated *in vitro* with *B*. *burgdorferi* (**Fig 1**). As expected, *Mir155* was not detectible by qRT-PCR in Mir155^-/-^ and DKO BMDMs, and cells lacking IL-10 had elevated levels of *Mir155* upon stimulation, compared to B6 (**Fig 1A**), which was shown previously [22].

To determine the effect of miR-155 and IL-10 on macrophage phagocytosis activity, peritoneal macrophages were incubated with a strain of GFP-expressing *B*. *burgdorferi* for 1 or 2 hours and quantified intracellular localization of *B*. *burgdorferi* in cells by flow cytometry (**Fig 1B)**, as done previously [21, 31]. We found that similar percentages of B6 and Mir155^-/-^ macrophages were GFP positive, but Il10^-/-^ macrophages had nearly double the percent of GFP-positive cells, indicative of higher phagocytic activity. This was largely dependent on miR-155, since DKO macrophages had nearly wild-type levels of phagocytic activity.

In BMDMs, IFN-inducible T cell chemokines *Cxcl9* and *Cxcl10* showed both IL-10-dependent and miR-155-dependent effects on expression (**Fig 1C**). Compared to B6 BMDMs, Il10^-/-^ cells had approximately 4-fold and 5-fold higher expression of *Cxcl9* and *Cxcl10*, respectively, upon stimulation with *B*. *burgdorferi* for 24 hours. In contrast, Mir155^-/-^ BMDMs tended to have lower expression levels of these two genes, compared to B6 BMDMs. Stimulated DKO BMDMs had intermediate levels of *Cxcl9* and *Cxcl10* expression, near or modestly above B6 levels, indicating that the effect of IL-10 and miR-155 were largely off-set when both of these regulators were absent.

IL-10 and miR-155 also had off-setting effects on myeloid cell activity as measured by cytokines secreted into the culture supernatant (**Fig 1D**). Il10^-/-^ BMDMs stimulated with *B*. *burgdorferi* had significantly higher levels of TNFα, IL-1β, IL-6, and IL-12 in culture supernatant, compared to supernatant from B6 BMDMs. Conversely, stimulated Mir155^-/-^ BMDMs tended to produce lower amounts of these innate cytokines, compared to B6 BMDMs. Stimulated DKO BMDM culture supernatant had significantly higher amounts of TNFα and IL-6, compared to B6 BMDM supernatant, but had lower levels of IL-6 and IL-12, compared to Il10^-/-^ BMDM supernatant. TNFα and IL-1β production detected in DKO supernatant were not significantly different than Il10^-/-^ BMDM supernatant levels, although both trended lower. These data show that immune activation (via miR-155) and immune suppression (via IL-10) have partially counteracting effects in macrophages stimulated with *B*. *burgdorferi*, consistent with the proposed hypothesis. These results are also similar to published results using Myd88^-/-^ and Tlr2^-/-^ BMDMs, which exhibit a defect in NF-κB activation and cytokine production in response to stimulation with *B*. *burgdorferi* sonicate [30].

### MicroRNA-155 and IL-10 are both required for suppression of Lyme carditis

To assess the impact of elevated *Mir155* expression levels in infected B6 heart tissue on carditis (**Table 1**), we infected B6, Mir155^-/-^, Il10^-/-^, and DKO mice with *B*. *burgdorferi* and measured Lyme carditis severity by blinded histopathology scoring at 2 and 3 weeks post-infection, periods reported to display peak disease (**Table 2**).

**Table 2:** Lyme carditis severity in 4 strains.

Strain	Overall lesion	PMN infiltrate	Mononucl infiltrate	Vasculitis severity	Total score
**2 weeks**					
B6	1.4 (±0.2)	0.6 (±0.1)	0.6 (±0.1)	0.6 (±0.2)	3.0 (±0.4)
Mir155^-/-^	2.0 (±0.3)	**1.8 (±0.3)**	1.4 (±0.2)	**1.6 (±0.2)**	**6.8 (±1.0)**
Il10^-/-^	**4.6 (±0.2)**	**4.6 (±0.2)**	**3.9 (±0.2)**	**4.4 (±0.2)**	**17.3 (±0.5)**
DKO	**3.8 (±0.5)**	**3.7 (±0.6)**	**3.7 (±0.6)**	**3.7 (±0.6)**	**14.9 (±2.2)**
**3 weeks**					
B6	0.6 (±0.2)	0.6 (±0.2)	0.9 (±0.1)	1.1 (±0.2)	3.9 (±0.3)
Mir155^-/-^	**1.6 (±0.2)**	**1.1 (±0.1)**	1.1 (±0.1)	**2.1 (±0.1)**	**6.3 (±0.1)**
Il10^-/-^	**4.4 (±0.2)**	**3.2 (±0.1)**	**3.2 (±0.1)**	**3.4 (±0.2)**	**13.2 (±0.5)**
DKO	**3.7 (±0.6)**	**2.5 (±0.3)**	**2.5 (±0.3)**	**3.4 (±0.3)**	**11.8 (±1.1)**

Histopathology scores of hearts of B6, Mir155^-/-^, Il10^-/-^, and DKO mice infected with *B*. *burgdorferi* for 2 or 3 weeks. Scores from 0–5 were assigned each sample with 5 being the most severe. Values shown are the mean (±SEM) scores. Total score is the sum of scores from each category. Bolded numbers indicate statistically significant differences between B6 and KO strain, as determined by ANOVA and Fisher’s LSD post-hoc analysis (p<0.05, n = 5 mice per group at each time point).

**Fig 2:**
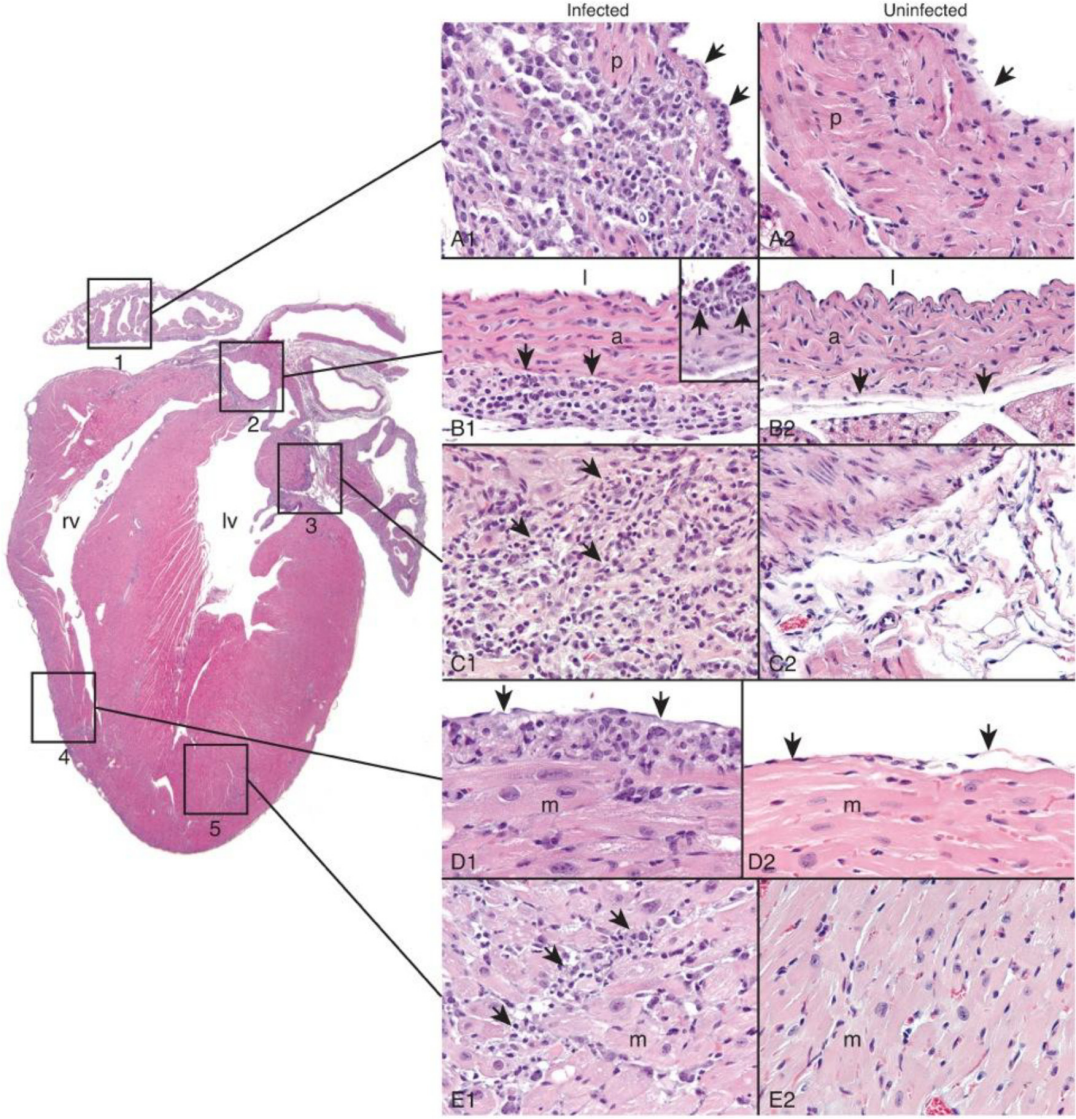
Description of Lyme carditis pathology. **A**, subacute vasculitis/perivasculitis induced by *B*. *burgdorferi* occurs in 5 distinct locations (pancarditis) in the sub-gross image (left-side of figure) of the heart (Box 1: atrium/auricle; Box 2: muscular artery; Box 3: vascularized connective tissue at the base of the heart; Box 4: epicardium and myocardium of the right ventricle; Box 5: myocardium of the left ventricle) (rv = right ventricle; lv = left ventricle). The interaction with, and injury caused by, the spirochete elicits a subacute inflammatory response that consists predominately of an admixture of neutrophils, lymphocytes, and monocytes/macrophages (i.e., histiocytes), as well as much smaller numbers of other mononuclear inflammatory cells (potentially plasma cells and/or dendritic cells). **A1**, pectinate muscle (p) and reactive endothelium (arrows) of atria/auricles contain subacute inflammatory cells (from Mir155^-/-^, Il10^-/-^, and DKO mouse strains); **A2,** similar area from a control animal (endothelium [arrow]); **B1**, vasa vasorum (arrows) of muscular arteries (a) contains similar inflammatory cells (l = lumen of muscular artery) (from Mir155^-/-^, Il10 ^-/-^, and DKO mouse strains). **B1 inset**, subintimal areas (arrows) of muscular arteries contain similar inflammatory cells. **B2**, similar area of a muscular artery (a) from a control animal (vasa vasorum [arrows]) (l = lumen of muscular artery); **C1**, stromal tissues at the base of the heart where blood vessels exit and enter the ventricles and atria/auricles, respectively (from Mir155^-/-^, Il10 ^-/-^, and DKO mouse strains) contain inflammatory cells (arrows). **C2**, similar area from a control animal; **D1**, loose connective tissue of the epicardium contains similar inflammatory cells (arrows) (from Mir155^-/-^, Il10 ^-/-^, and DKO mouse strains). The cardiac myocytes (myocardium [m]) are reactive and enlarged. **D2**, similar area of the epicardium (arrows) from a control animal (myocardium [m]); and **E1**, myocardium (m) of the ventricles contains similar inflammatory cells (arrows) (from Mir155^-/-^, Il10 ^-/-^, and DKO mouse strains). The cardiac myocytes are reactive and enlarged. **E2**, similar area of the ventricular myocardium (m) from a control animal. H&E stains, images representative of two infection experiments, one at 2 and one at 3 weeks post-infection (n = 5 mice per strain for each infection).

The primary lesion in all strains of mice used in this study was subacute vasculitis/perivasculitis characterized by inflammation of and around the microvasculature of one or more types of heart tissues (pancarditis) as described below. Vascular endothelium appeared to be a primary target for infection by *B*. *burgdorferi* within these tissues. The inflammatory response consisted predominately of an admixture of neutrophils and mononuclear cells such as lymphocytes and monocytes/macrophages (**Table 2**). Other types of mononuclear cells in the inflammatory exudate may have included small numbers of plasma cells and other types of mononuclear cells (i.e., dendritic cells) that cannot be differentiated based on cellular morphology. Vasculitis/perivasculitis occurred in 5 distinct locations in the heart (pancarditis) (**Fig 2**): 1) in vascularized interstitium of the pectinate muscle (i.e., myocarditis) and subendothelial areas of atria/auricles; 2) in the vasa vasorum and subintimal areas of muscular arteries (i.e., arteritis); 3) in vascularized stromal tissues at the base of the heart where blood vessels exit and enter the ventricles and atria/auricles, respectively; 4) in the vascularized loose connective tissue of the epicardium (i.e., epicarditis); and 5) in vascularized interstitium of cardiac muscle of the ventricles (i.e., myocarditis).

**Fig 3:**
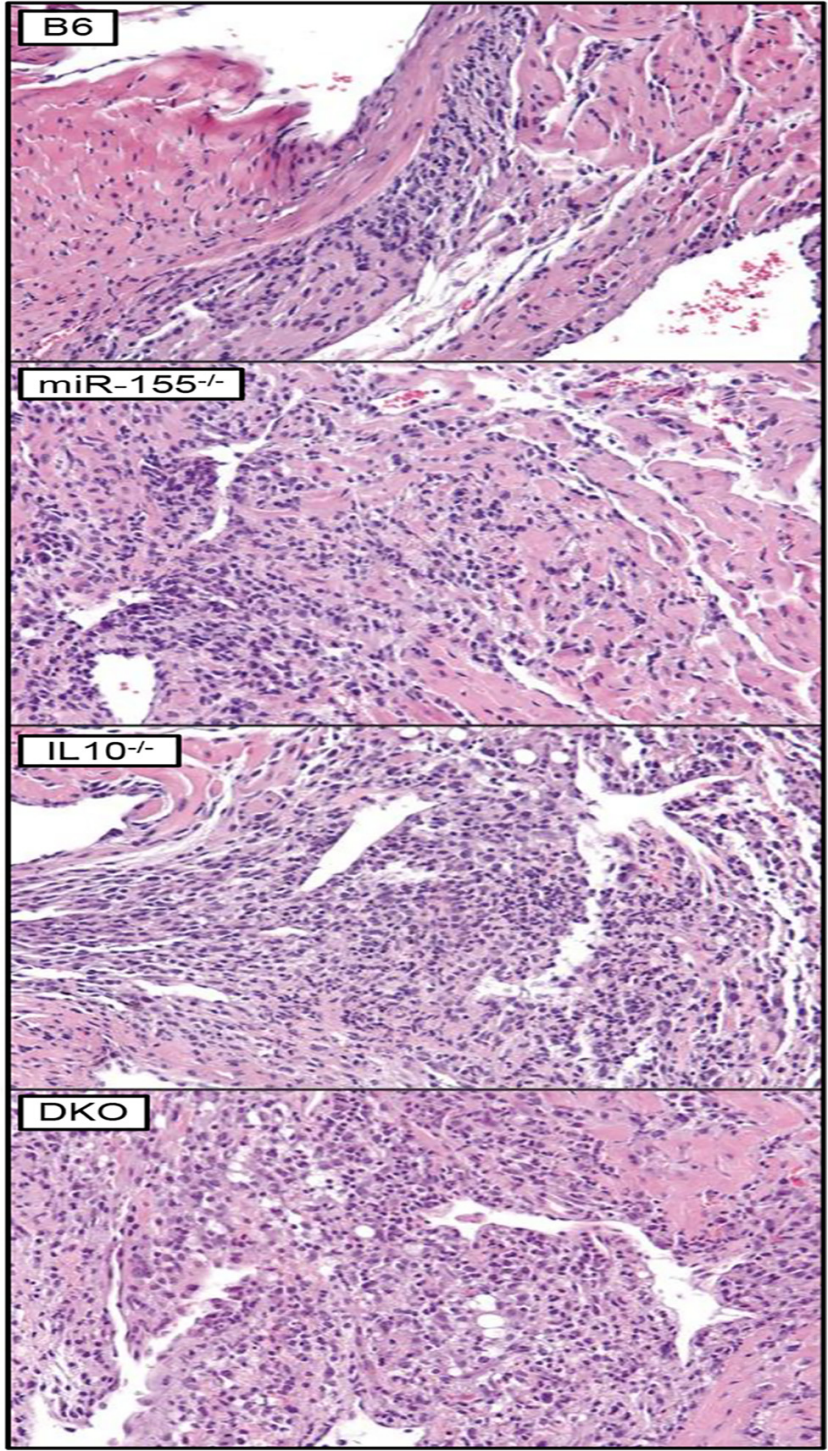
Lyme carditis severity is modulated by both miR-155 and IL-10. Comparison of the severity of subacute vasculitis/perivasculitis affecting stromal tissues at the base of the heart where blood vessels exit and enter the ventricles and atria/auricles, respectively between mouse strains at 2 weeks following infection with *B*. *burgdorferi*. The subacute inflammatory response consists predominately of an admixture of PMNs, lymphocytes, and monocytes/macrophages as well as much smaller numbers of other mononuclear inflammatory cells (potentially plasma cells and/or dendritic cells). H&E stains, images representative of two infection experiments, one at 2 and one at 3 weeks post-infection (n = 5 mice per strain for each infection).

Vasculitis/perivasculitis occurred at varying degrees of severity and location in the heart and severity was based on the strain of mouse infected with *B*. *burgdorferi*. All mouse strains (B6, Mir155^-/-^, Il10^-/-^, and DKO) had subacute vasculitis/perivasculitis of vascularized stromal tissues at the base of the heart (**Fig 3, Table 2**). However, whereas B6 mice had mild lesions, Mir155^-/-^, Il10^-/-^, and DKO mouse strains always had more severe lesions. Similarly, B6 mice also only had mild lesions of the muscular arteries and atria/auricles; whereas the other mouse strains developed moderate to severe vasculitis/perivasculitis in the other 4 areas of the heart listed above (**Table 2**). The morphologic characteristics of the inflammatory response appeared similar across all mouse strains.

These results suggested that *both* miR-155 and IL-10 were required for suppression of Lyme carditis, despite having opposing roles in immune response. Furthermore, disruption of the delicate balance between immune activation and suppression of inflammation maintained by this negative feedback network exacerbated carditis severity. Interestingly, previously published results using C3H and C3H Il10^-/-^ mice [36] indicate that the role of IL-10 in suppressing Lyme carditis may be strain-specific, since no differences were observed in carditis severity between C3H mice with or without IL-10.

### MicroRNA-155 has no significant effect on Lyme arthritis severity

It has been well established that Il10^-/-^ mice develop moderate to severe LA, which is associated with a strong localized IFNγ signature [11]. Expression of miR-155 is also associated with T cell-dependent inflammation [37]. We hypothesized that the inability of IL-10 to suppress miR-155 during Lyme arthritis development could be contributing to increased arthritis severity [21]. We therefore infected B6, Mir155^-/-^, Il10^-/-^, and DKO mice with *B*. *burgdorferi* for 4 weeks, after which we assessed arthritis development (**Table 3**). B6 mice developed mild arthritis, and Il10^-/-^ mice developed more severe arthritis, as measured by ankle swelling and histopathology scoring, as has been reported previously [11]. However, miR-155 appeared to have little effect on Lyme arthritis severity. Mir155^-/-^ mice had mild arthritis similar to B6 mice, and DKO mice had severe arthritis, similar to Il10^-/-^ mice. These results suggested that miR-155 is not required for severe arthritis development.

**Table 3:** Lyme arthritis severity in 4 strains.

Strain	Ankle swelling	Overall lesion	PMN Infil.	Mono Infil.	Sheath thickness	Reactive/Repar.	Total score
B6	0.29(±0.06)	1.7(±0.4)	1.1(±0.3)	0.2(±0.1)	1.4(±0.3)	1.0(±0.5)	5.3 (±1.3)
Mir155^-/-^	0.18(±0.04)	1.1(±0.3)	0.4(±0.1)	0.3(±0.1)	1.9(±0.2)	0.3(±0.1)	3.1 (±0.7)
Il10^-/-^	**0.72(±0.05)**	2.5(±0.3)	**2.1(±0.3)**	**1.3(±0.2)**	**2.3(±0.3)**	0.8(±0.4)	**8.9 (±1.0)**
DKO	**0.69(±0.06)**	2.7(±0.4)	**2.1(±0.5)**	**1.3(±0.3)**	**2.4(±0.4)**	2.0(±0.5)	**10.5(±1.7)**

Arthritis severity for B6, Mir155^-/-^, Il10^-/-^, and DKO mice was determined by swelling of rear ankles and by histopathology scoring. Blinded measurements of rear ankles were taken before infection and after 4 weeks of infection with *B*. *burgdorferi*. Ankle swelling values are the mean (±SEM) change in ankle diameter (in mm) after infection. Histopathology scores from 0 to 5, with 5 being most severe, were assigned to each sample. Total score is the sum of scores from each category. Bolded numbers indicate statistically significant differences between B6 and KO strain, as determined by ANOVA and Fisher’s LSD post-hoc analysis (p<0.05, n = 7–10 mice per group).

### MicroRNA-155 has a significant effect on host defense and B cell response

**Fig 4:**
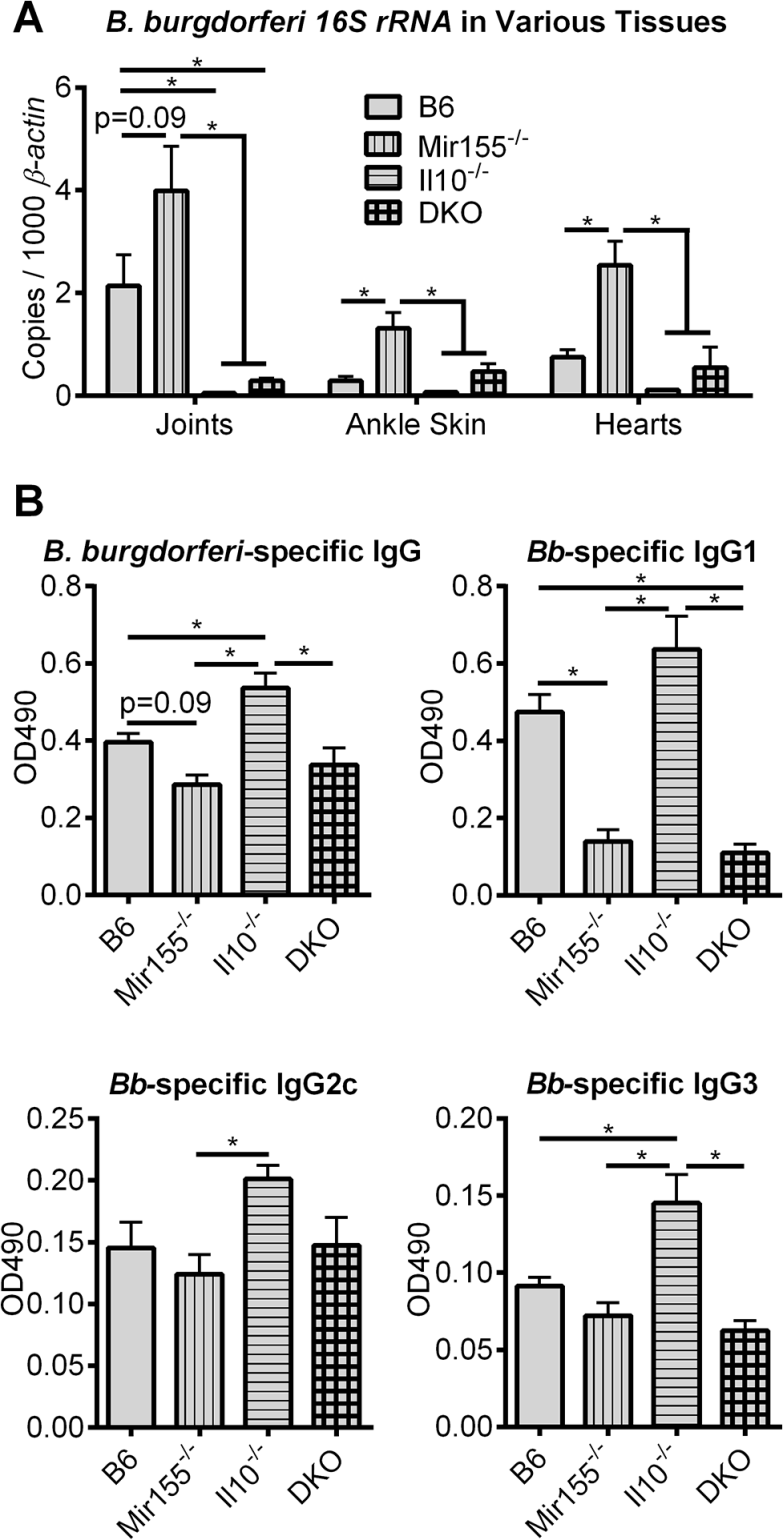
Host defense and B cell responses are modulated by the IL-10/miR-155 regulatory network. B6, Mir155^-/-^, Il10^-/-^ and DKO mice were infected with *B*. *burgdorferi* and host defense was assessed. **A,** joints were collected from mice infected for 4 weeks, and ankle skin and hearts were collected from mice infected for 3 weeks, and bacterial numbers were determined by measuring expression of *B*. *burgdorferi 16S rRNA*, compared to *β-actin*. **B,** *B*. *burgdorferi-*specific IgG (total), IgG1, IgG2c, and IgG3 were measured by ELISA in serum from mice infected for 4 weeks. Statistically significant differences between strains were determined by ANOVA (Tukey’s post-hoc), and are indicated (* p<0.05), with adjusted p-values included for trends not achieving statistical significance (p<0.1). Representative of 2 independent experiments at 4 weeks post-infection, and once at 3 weeks post-infection (n≥ 5 mice per strain for each experiment).

The finding that both miR-155 and IL-10 were required for suppression of Lyme carditis (**Table 2, Fig 3**), but not LA (**Table 3**), suggested that miR-155 upregulation in infected hearts (**Table 1**) might be an important element of immune activation and host defense specifically within cardiac tissue. We therefore assessed bacterial burden in various tissues from mice infected with *B*. *burgdorferi* for 3 weeks (hearts, ankle skin) or 4 weeks (joints) by measuring *B*. *burgdorferi 16S rRNA* expression by qRT-PCR (**Fig 4A**). As expected, Il10^-/-^ mice had the lowest bacterial numbers in all three tissues, consistent with a hyperactive immune response and elevated host defense. In contrast, miR-155^-/-^ mice had the greatest numbers of bacteria in all three tissues examined, consistent with impaired host defense activation upon infection. B6 and DKO mice had intermediate numbers of bacteria in infected tissue. Interestingly, while there were less than 2-fold (non-significant) differences between DKO and B6 mice in heart and ankle skin bacterial loads, joint bacterial numbers were approximately 7-fold lower (p<0.05) in DKO mice, compared to B6 mice. These data show that IL-10/miR-155-mediated immune regulation has tissue-specific effects on host defense. The differences in phagocytosis activity between the 4 strains (**Fig 1B**) revealed a similar pattern of heightened activity in IL-10 deficient macrophages, compared to B6 macrophages, with little impact of miR-155 deficiency, compared to B6 macrophages, pointing to a dominant impact of IL-10 in macrophage-mediated host defense to *B*. *burgdorferi*. In joints, miR-155 appeared to have only a modest effect on bacterial numbers, and IL-10 had the greatest effect on host defense. In ankle skin and heart tissue, however, both IL-10 and miR-155 had a dramatic impact on bacterial numbers, and the counteracting effects were off-set in the DKO mice. These results are consistent with the important role of MyD88/ NF-κB-dependent cytokines in defense against *B*. *burgdorferi*, and the role of miR-155 in modulating cytokine expression **(Fig 1)**.

The results from **Fig 4A**, along with differences in tissue-specific pathologies (**Tables 2 and 3**), suggested the miR-155/IL-10 network was regulating multiple aspects of immune response and host defense. *B*. *burgdorferi* is a potent B cell mitogen, and infection triggers a robust antibody response, which is enhanced in Il10^-/-^ mice [12]. Conversely, mice lacking miR-155 have defects in antibody isotype switching [25], in germinal center formation [26], and in T_fh_ function [38]. These opposing effects of IL-10 and miR-155 on humoral response led to an assessment of the contribution of these regulators to antibody production during infection with *B*. *burgdorferi*. Mice were infected for 4 weeks and the relative abundance of *B*. *burgdorferi*-specific antibody isotypes were determined by ELISA (**Fig 4B**). Consistent with previous studies, *B*. *burgdorferi*-specific IgG levels trended lower in Mir155^-/-^ mice (p = 0.09), and were greater in Il10^-/-^ mice, compared to B6 IgG levels. IgG levels in DKO mice were very similar to IgG levels in Mir155^-/-^ mice. Interestingly, *B*. *burgdorferi*-specific IgG1 was not detectible above background in either Mir155^-/-^ or DKO mice. IgG2c and IgG3 were detected in all 4 strains, but were highest in Il10^-/-^ mice, and Mir155^-/-^ and DKO mice had IgG2c and IgG3 levels similar to B6 IgG2c and IgG3. These data show that the elevated antibody levels observed in IL10^-/-^ mice are dependent upon miR-155, and that miR-155 is required for IgG1 isotype switching. Interestingly, a *B*. *burgdorferi-*specific IgG deficiency is observed in Myd88^-/-^ mice [30], and MyD88 has been shown to be important in antibody class switching and humoral immunity [39]. Consistent with these results, two validated targets of miR-155, SHIP1 [40] and SOCS1 [41], are negative regulators of MyD88 signaling.

### Effects of microRNA-155 and IL-10 on Th1 and innate cytokine responses in infected tissue and serum

Histopathology results from hearts (**Table 2**) and joints (**Table 3**) of infected mice indicated that miR-155 and IL-10 were having effects on immune responses and host defense in a tissue-specific manner. We were curious about what effect these two immune regulators were having on cytokine expression within these tissues and systemically, particularly in light of the role of IL-10 in down-modulating pro-inflammatory cytokine function, and the role of miR-155 in potentiating cytokine production.

To identify cytokines influenced by miR-155 and IL-10 in heart tissue, we infected B6, Mir155^-/-^, Il10^-/-^, and DKO mice with *B*. *burgdorferi* and collected hearts at 3 weeks post-infection for cytokine expression analysis (**Table 4**). We found that *Ifng* was significantly upregulated in hearts of all 4 strains at 3 weeks post-infection, compared to uninfected controls. This adaptive cytokine has been shown previously to be upregulated during Lyme carditis in mice [42], and IFNγ production by invariant NKT cells is important in host defense [16]. While all 4 strains showed upregulation of *Ifng*, expression in infected Mir155^-/-^ hearts was lower than expression in infected B6 hearts (p = 0.04 by Student t-test), although this difference lost statistical significance by ANOVA In infected Il10^-/-^ and DKO hearts, *Ifng* expression was significantly higher than in both B6 and Mir155^-/-^ hearts. Il10^-/-^ and DKO mice also had significant upregulation of *Cxcl10* and *Il12* in infected heart tissue, compared to uninfected controls, which were not observed in infected B6 and Mir155^-/-^ hearts, indicating an elevated Th1 response in infected hearts of mice lacking IL-10 (Il10^-/-^ and DKO mice). These results are consistent with our proposed hypothesis, and may be due to differences in STAT1 activation, a protein important in protection against Lyme carditis [15].

**Table 4:** Expression of Th1 and innate cytokines in hearts of infected mice.

**Th1 cytokines**						
**Strain**	***Ifng***		***Il12***		***Cxcl10***	
	**Control**	**Infected**	**Control**	**Infected**	**Control**	**Infected**
B6	0.2 (±0.04)	*0*.*8 (±0*.*1)*	1.9 (±0.7)	1.7 (±0.1)	3.3 (±0.3)	2.3 (±0.3)
Mir155^-/-^	0.08 (±0.04)	*0*.*5 (±0*.*09)*	1.5 (±0.4)	1.6 (±0.4)	1.7 (±0.4)	2.2 (±0.2)
Il10^-/-^	0.8 (±0.3)	***10*.*7 (±1*.*0)***	1.7 (±0.5)	***10*.*7 (±2)***	1.4 (±0.1)	***25 (±5*.*5)***
DKO	0.3 (±0.06)	***11*.*6 (±0*.*8)***	1.5 (±0.4)	***8*.*9 (±2*.*2)***	1.6 (±0.4)	***22 (±3*.*2)***
**Innate cytokines**						
**Strain**	***Il6***		***Il1b***		***Tnfa***	
	**Control**	**Infected**	**Control**	**Infected**	**Control**	**Infected**
B6	1.3 (±0.3)	0.4 (±0.1)	1.8 (±0.4)	1.1 (±0.1)	0.6 (±0.3)	0.8 (±0.1)
Mir155^-/-^	0.6 (±0.1)	0.8 (±0.2)	0.8 (±0.1)	1.9 (±0.5)	0.4 (±0.04)	0.9 (±0.3)
Il10^-/-^	1.0 (±0.6)	*2*.*0 (±0*.*1)*	1.0 (±0.3)	*4.*5 (±0*.*4)*	0.6 (±0.3)	***1*.*9 (±0*.*4)***
DKO	0.2 (±0.1)	*7.*0 (±2*.*0)*	1.9 (±0.3)	***2*.*8 (±0*.*3)***	0.5 (±0.1)	*1*.*4 (±0*.*1)*

Mean (±SEM) number of transcripts, relative to *β-actin*, of cytokines from hearts of uninfected control mice and mice infected with *B*. *burgdorferi* for 3 weeks. Italicized numbers indicate statistically significant differences in transcripts between uninfected and infected mice of the same strain, as determined by Student’s t-test (p<0.05). Bold numbers indicate statistically significant differences in transcripts between infected Il10^-/-^ or DKO strains, compared to infected B6 and Mir155^-/-^ strains, and * indicates statistically significant differences in transcripts between infected between Il10^-/-^ and DKO strains, as determined by ANOVA, followed by Tukey’s post-hoc analysis (p<0.05, n = 6 mice per group).

Innate cytokines also showed some degree of dysregulation in hearts of infected Il10^-/-^ and DKO mice. *Il6* was significantly elevated in infected hearts of DKO mice, compared to the other three strains (although *Il6* was upregulated in hearts of infected Il10^-/-^ mice, compared to uninfected Il10^-/-^ hearts). *Il1b* was higher in hearts of Il10^-/-^ mice compared to the other three strains, as well as in hearts of DKO mice, compared to B6 and Mir155^-/-^ hearts. *Tnfa* was modestly elevated in hearts of Il10^*-/-*^ mice, compared to the other three strains (although *Tnfa* was also upregulated in DKO hearts upon infection, compared to uninfected DKO hearts). These results show that while IL-10 appears to be more important than miR-155 in regulating IFNγ production and a Th1 response, miR-155 and IL-10 are both important in proper regulation of innate response cytokines and acute inflammation. Indeed, data shown in **Fig 1** support the idea that lack of miR-155 in macrophages has a profound effect on innate immune activation in response to *B*. *burgdorferi*, and this could explain why higher bacterial numbers were seen in infected Mir155^-/-^ hearts (**Fig 4A**). Histopathology analysis (**Table 2**) suggests that defects in myeloid cell activation, and not recruitment, are responsible for increased bacterial numbers, since PMN infiltrate was significantly higher in hearts of infected Mir155^-/-^ mice, compared to infected B6 mouse hearts. Importantly, these results show that maintaining a proper balance between host defense and innate and adaptive cellular responses by miR-155 and IL-10 appear to be critical in limiting Lyme carditis. Hypo-activation of host response to infection can lead to increased bacterial burden and increased carditis in the Mir155^-/-^ mouse, and hyper-activation of both innate and adaptive responses can lead to excess inflammation and carditis in Il10^-/-^ and DKO mice.

Although miR-155 did not appear to have an effect on Lyme arthritis (in contrast to Lyme carditis), we were curious to determine what effects miR-155 and IL-10 had on cytokine expression in joints of mice infected with *B*. *burgdorferi* for 4 weeks. At 4 weeks post-infection, Th1-associated *Ifng*, *Cxcl10*, and *Il12* transcript levels were significantly higher in joints of Il10^-/-^ and DKO mice, compared to transcript levels in joints of B6 and Mir155^-/-^ mice (**Table 5**). Curiously, *Cxcl10* expression levels were highest in joints of infected DKO mice, compared to the other three mouse strains, and *Il6* expression was highest in joints of infected Il10^-/-^ mice, compared to the other three strains. *Il6* was also upregulated in infected DKO mouse joints, compared to joints from uninfected DKO mice, but did not achieve statistical significance by ANOVA when compared to joints of B6 or Mir155^-/-^ mice. Also, while *Ifng* levels were not significantly elevated in infected B6 and Mir155^-/-^ mouse joints, compared to uninfected controls, the IFNγ responsive chemokine *Cxcl10* showed significant upregulation in these two mouse strains upon infection, although the degree of upregulation trended lower in Mir155^-/-^ joints compared to B6 joints (p = 0.001 by Student t-test, not significant by ANOVA). *Il1b* showed modest upregulation in joints of Il10^-/-^ and DKO mice upon infection, compared to uninfected controls, but differences between these strains and joints of infected B6 and Mir155^-/-^ mice did not achieve statistical significance by ANOVA. *Tnfa* was not significantly upregulated in any strain, compared to uninfected controls. This may be due to the relatively late time point (4 weeks), by which time the acute response has subsided.

**Table 5:** Expression of cytokines in joints of infected mice.

**Th1 cytokines**						
**Strain**	***Ifng***		***Il12***		***Cxcl10***	
	**Control**	**Infected**	**Control**	**Infected**	**Control**	**Infected**
B6	0.09 (±0.01)	0.17 (±0.03)	0.9 (±0.2)	1.5 (±0.4)	0.6 (±0.1)	*2*.*9 (±0*.*2)*
Mir155^-/-^	0.07 (±0.03)	0.15 (±0.04)	0.8 (±0.4)	0.9 (±0.1)	0.7 (±0.1)	*1*.*7 (±0*.*2)*
Il10^-/-^	0.08 (±0.01)	***3*.*5 (±0*.*5)***	1.0 (±0.2)	***8*.*4 (±1*.*7)***	0.6 (±0.1)	***35*.*1 (±5)***
DKO	0.16 (±0.04)	***4*.*8 (±0*.*7)***	0.9 (±0.3)	***6*.*8 (±0*.*9)***	1.2 (±0.1)	*52.*4 (±8)*
**Innate cytokines**						
**Strain**	***Il6***		***Il1b***		***Tnfa***	
	**Control**	**Infected**	**Control**	**Infected**	**Control**	**Infected**
B6	0.8 (±0.3)	1.0 (±0.2)	1.0 (±0.1)	1.0 (±0.2)	0.2 (±0.03)	0.2 (±0.04)
Mir155^-/-^	1.1 (±0.2)	0.7 (±0.2)	0.6 (±0.1)	0.8 (±0.1)	0.2 (±0.04)	0.2 (±0.05)
Il10^-/-^	1.1 (±0.6)	**4.6 (±1.8)**	0.6 (±0.2)	*1*.*6 (±0*.*3)*	0.3 (±0.01)	0.4 (±0.06)
DKO	0.3 (±0.1)	*3*.*5 (±0*.*6)*	0.6 (±0.1)	*1*.*6 (±0*.*3)*	0.2 (±0.02)	0.3 (±0.06)

Mean (±SEM) number of transcripts, relative to *β-actin*, of cytokines from joints of uninfected control mice and mice infected with *B*. *burgdorferi* for 4 weeks. Italicized numbers indicate statistically significant differences in transcripts between uninfected and infected mice of the same strain, as determined by Student’s t-test (p<0.05). Bold numbers indicate statistically significant differences in transcripts between infected Il10^-/-^ or DKO strains, compared to infected B6 and Mir155^-/-^ strains, and * indicates statistically significant differences in transcripts between infected between Il10^-/-^ and DKO strains, as determined by ANOVA, followed by Tukey’s post-hoc analysis (p<0.05, n = 8–10 mice per group).

**Fig 5:**
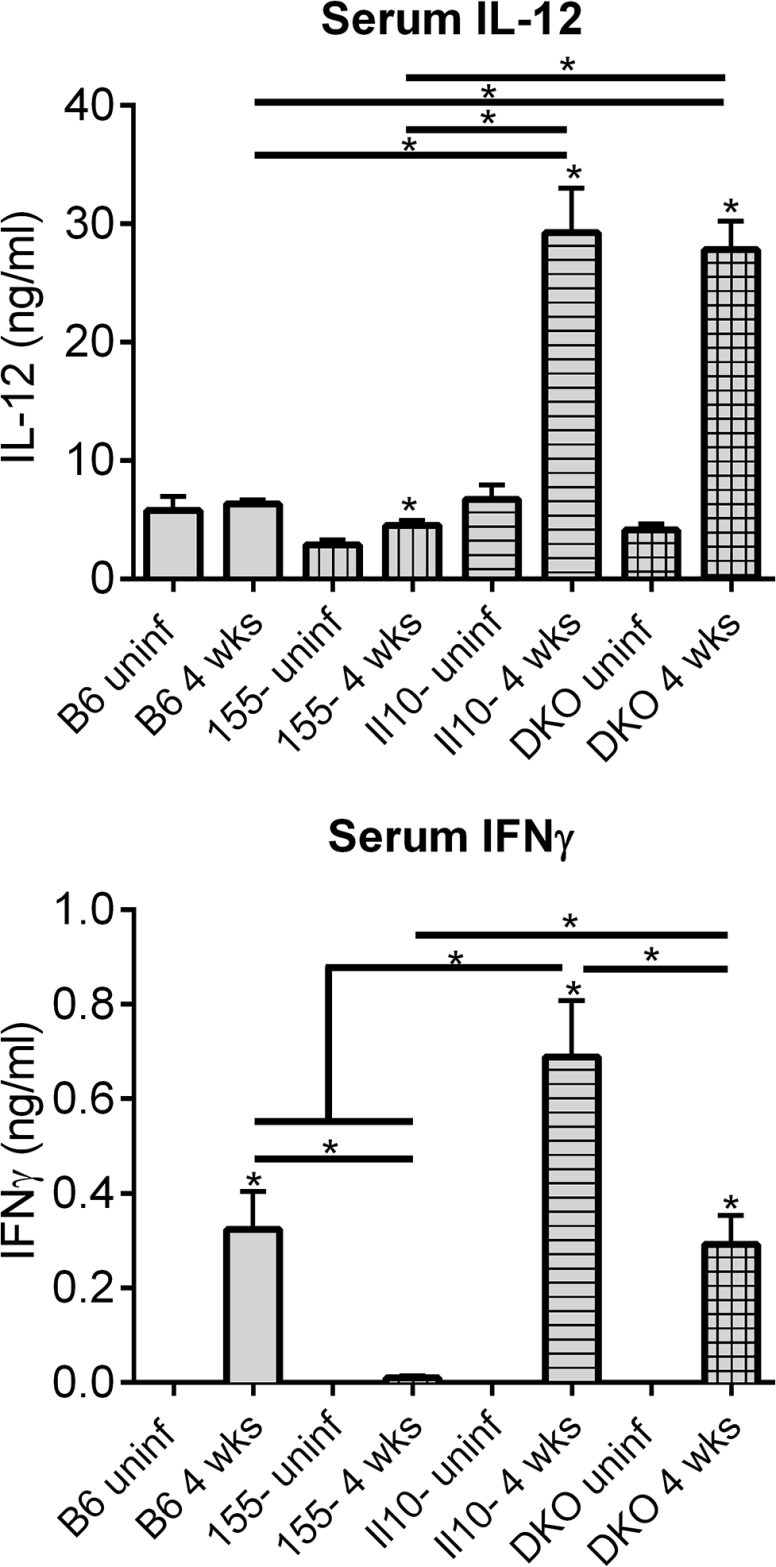
IL-12 and IFNγ levels in serum of mice infected for 4 weeks. Cytokines were measured by ELISA in serum collected from B6, Mir155^-/-^, Il10^-/-^ and DKO mice infected with *B*. *burgdorferi* for 4 weeks. Statistically significant differences between cytokine levels before and after infection for each strain were determined by Student t-test, and between infected strains by ANOVA (Tukey’s post-hoc), and are indicated (* p<0.05, n≥ 8 mice per strain).

The most striking results from **Tables 4 & 5** were very strong upregulation of Th1 cytokines in infected hearts and joints of Il10^-/-^ and DKO mice, compared to uninfected tissue, and this upregulation was reduced or absent in hearts and joints of B6 and Mir155^-/-^ mice, compared to uninfected tissue. However, some cytokines are post-transcriptionally regulated. To determine whether this robust Th1 profile observed by transcript analysis in tissue was also observed systemically by protein analysis, we measured IL-12 and IFNγ cytokine levels in serum of mice at 4 weeks post-infection (**Fig 5**). Serum levels of the classic Th1 cytokine IL-12 in infected Il10^-/-^ and DKO mice were elevated compared to uninfected controls, and were significantly higher than IL-12 levels in serum of infected B6 and Mir155^-/-^ mice. Interestingly, serum from infected Mir155^-/-^ mice had very low IFNγ levels, compared to serum from the other three strains at 4 weeks post-infection, and infected Il10^-/-^ mouse serum had very high IFNγ levels, compared to serum from the other three strains. Serum from infected B6 and DKO mice had intermediate and very similar IFNγ levels. These observations were somewhat different than those seen in tissue by mRNA analysis, and could be the result of tissue-specific vs. systemic differences in regulation of IFNγ, either at the transcriptional or post-transcriptional level, and further research will be required to elucidate differences between strains.

## Discussion

**Fig 6:**
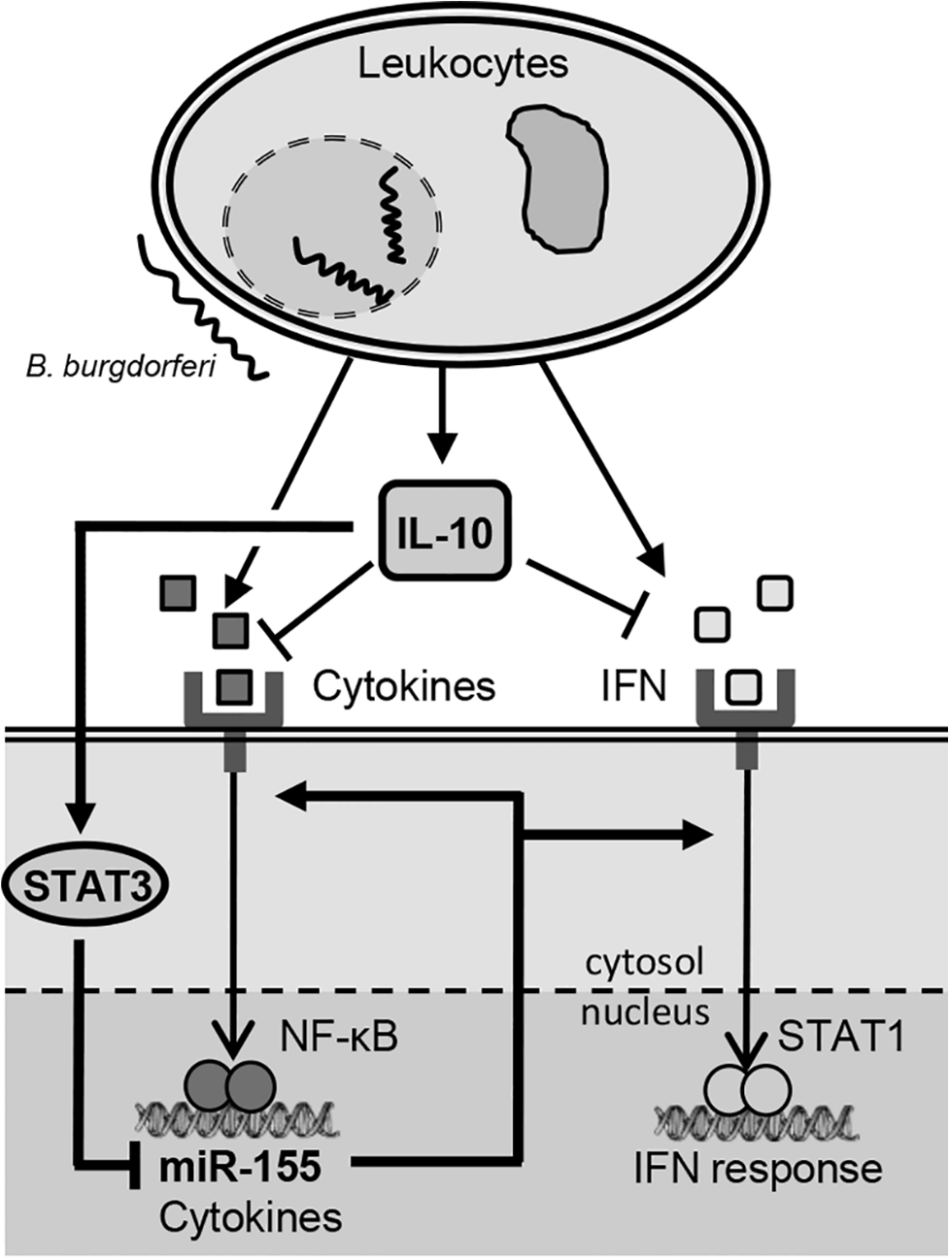
Proposed model: antagonistic interplay between IL-10 and miR-155 modulates immune response during infection with *B*. *burgdorferi*. Host immune cells, such as leukocytes, secrete various innate and/or adaptive cytokines at the site of *B*. *burgdorferi* infection. IL-10 is also secreted, which acts to limit inflammation by suppressing pro-inflammatory cytokine activity. One mechanism of IL-10 immune modulation involves STAT3-dependent suppression of expression of miR-155, which is upregulated during infection through NF-κB activation, and which enhances cytokine signaling (such as JAK-STAT signaling and NF-κB signaling) and immune activation (IL-10/miR-155 regulatory circuit in bold).

The immune system has multiple means whereby a balance between immune activation and down-regulation is achieved, facilitating effective defense against pathogens and quickly returning to homeostasis following infection. MicroRNAs often act in the context of regulatory networks to confer robustness to biological processes [43]. In this study, we explored how one such regulatory network, IL-10-mediated regulation of miR-155 [22], influences Lyme disease pathogenesis, immune activation, and host defense during infection with *B*. *burgdorferi* (**Fig 6**).

IL-10 has been called the master regulator of immunity to infection [23]. However, many aspects of the mechanism of IL-10-mediated immune regulation remain unknown. Our findings showed that regulation of miR-155 expression is an important means whereby IL-10 controls immune activation. MiR-155 is involved in modulating many immune response pathways [19, 44, 45] through targeting hundreds of genes in myeloid cells [46], B cells [47], and CD4+ T cells [48], and it is likely that IL-10-mediated suppression of miR-155 is important in regulating many cellular processes and genes in many cell types. We were therefore curious whether the very high expression of miR-155 observed in infected Il10^-/-^ mice (**Table 1**) [21] was of biological relevance.

While young Mir155^-/-^ mice do not exhibit overt defects in development [25], mice constitutively over-expressing miR-155 spontaneously develop chronic inflammation [46] and leukemia [49]. Additionally, *chronic* dysregulation of miR-155 in T_fh_ cells contributes to low-grade inflammation and autoimmunity [38]. Here, we showed that *acute* dysregulation of miR-155 during *B*. *burgdorferi* infection in Il10^-/-^ mice contributes to myeloid cell hyper-activation (**Fig 1**), elevated antibody production and altered isotype switching (**Fig 4B**), and higher serum IFNγ levels (**Fig 5**). We also showed that both miR-155 and IL-10 are important for protection against heart inflammation and carditis (**Figs 2 and 3, Tables 2 and 4**), and that LA development and Th1 responses are predominantly mediated by miR-155-independent effects of IL-10 (**Tables 3–5**).

Our findings highlight the protective role of both miR-155 and IL-10 in limiting Lyme carditis. This is clinically relevant, particularly in light of recently reported cases of deaths associated with post-mortem identification of high numbers of spirochetes in heart tissue [5]. These results indicate that Lyme carditis is sensitive to both hypo-activation and hyper-activation of immune responses, but LA severity is dependent largely upon degree of inflammation. Lyme carditis in humans is an early systemic manifestation of disease [3], where bacterial numbers and acute inflammation may play a greater role in influencing disease severity. In these cases, miRs such as miR-155 may have an important role in modulating immune activation.

Innate immune regulators are absolutely required for effective control of spirochete infection and immune activation. This has been shown in mice (Myd88^-/-^ [50] and Tlr2^-/-^ [51] mice have significant immune and host defense defects) as well as in humans (TLR2 expression [52] and a Tlr1 polymorphism [7] are associated with altered immune response to *B*. *burgdorferi* OspA vaccination and *B*. *burgdorferi* RST1 infection, respectively). In some aspects, the Mir155^-/-^ mouse is similar to Myd88^-/-^ and Tlr2^-/-^ mice, albeit with a less severe phenotype. All three knockout strains show varying degrees of hypo-active immune activation and all three show defects in host defense. However, unlike Myd88^-/-^ and Tlr2^-/-^ mice, Mir155^-/-^ mice still retain intact signaling pathways, and only the amplitude of the signal is altered.

It is unknown which miR-155 targets and pathways are most relevant to our observations, but it is likely that this miR has a cumulative effect on immunity and infection. Recent published findings have shown that miR-155 regulation of AID is important for IL-10-mediated regulation of B cell activation [53]. Also, SHIP1 was shown to be repressed by miR-155 in BMDMs [40], which is reversed with the addition of IL-10 [22]. In T cells, the T_reg_-specific transcription factor Foxp3 regulates miR-155 expression and its target SOCS1, thereby maintaining homeostasis [54]; and miR-155 in T_fh_ cells regulates NF-κB, AP1, and mTOR signaling pathways, influencing development and function [38]. Importantly, these data, along with previously published results, confirm that the IL-10/miR-155 network regulates several immunological processes, that there are important differences between LA and carditis pathogenesis, and that IL-10-mediated control of miR-155 expression is required for maintaining healthy immunity during *B*. *burgdorferi* infection.

## Supporting Information

S1 ARRIVE Checklist ARRIVE Guidelines Checklist. Based on recommendations for reporting in vivo experiments from the National Centre for the Replacement Refinement & Reduction of Animals in Research (NC3R).
(PDF)
